# Uncemented or cemented stems in first-time revision total hip replacement? An observational study of 867 patients including assessment of femoral bone defect size

**DOI:** 10.1080/17453674.2020.1846956

**Published:** 2020-11-12

**Authors:** Yosef Tyson, Christer Hillman, Norbert Majenburg, Olof Sköldenberg, Ola Rolfson, Johan Kärrholm, Maziar Mohaddes, Nils P Hailer

**Affiliations:** a Section of Orthopaedics, Department of Surgical Sciences, Uppsala University Hospital, Uppsala, Sweden;; b The Swedish Hip Arthroplasty Register, Gothenburg, Sweden;; c Department of Orthopaedics, Danderyd University Hospital Corp, Stockholm, Sweden;; d Department of Clinical Sciences, Karolinska Institutet, Danderyd Hospital, Division of Orthopaedics, Stockholm, Sweden;; e University of Groningen, Groningen, The Netherlands;;; f Department of Orthopaedics, Institute of Clinical Sciences, Sahlgrenska Academy, Gothenburg University, Gothenburg, Sweden

## Abstract

Background and purpose — Uncemented stems are gradually replacing cemented stems in hip revision surgery. We compared the risk of re-revision between uncemented and cemented revision stems and assessed whether the different fixation methods are used in similar femoral bone defects.

Patients and methods — 867 patients operated on with uncemented or cemented stems in first-time hip revision surgery due to aseptic loosening performed 2006–2016 were identified in the Swedish Hip Arthroplasty Register. Preoperative femoral bone defect size was assessed on radiographs of all patients. Cox regression models were fitted to estimate the adjusted risk of re-revision during different postoperative time periods. Re-revision of any component for any reason, and stem re-revision, as well as risk of cause-specific re-revision was estimated.

Results — Most patients in both fixation groups had Paprosky class IIIA femoral bone defects prior to surgery, but there were more severe bone defects in the cemented group. The adjusted risk of re-revision of any component for any reason was higher in patients with uncemented compared with those with cemented revision stems during the first 3 years after index surgery (hazard ratio [HR] 4, 95% confidence interval [CI] 2–9). From the 4th year onward, the risk of re-revision of any component for any reason was similar (HR 0.5, CI 0.2–1.4). Uncemented revision stems conferred a higher risk of dislocation compared with cemented stems (HR 5, CI 1.2–23) during the first 3 years.

Interpretation — Although not predominantly used in more complex femoral defects, uncemented revision stem fixation confers a slightly higher risk of re-revision during the first years, but this risk is attenuated after longer follow-up.

The increased use of primary total hip replacement (THR) has been followed by a steady rise in the frequency of revision surgery (Kurtz et al. 2007, Rajaee et al. 2018), and the use of uncemented revision stems is increasing in most countries (Swedish Hip Arthroplasty Register [SHAR] 2015). Some surgeons consider uncemented revision stems to be more appropriate in situations with extensive femoral bone loss, but others use long cemented revision stems, sometimes in conjunction with bone impaction grafting. Ultimately, the choice of fixation method in revision surgery is a matter not only of science and evidence, but also of taste and local tradition.

Register-based studies indicate that uncemented revision stems may have inferior implant survival when compared with cemented stems, especially in the older population (Weiss et al. 2011, Tyson et al. 2019). However, these register studies lack information on femoral bone defect size, a factor that can affect the choice both of fixation method and of outcome in terms of re-revision rates (Paprosky et al. 1999, Pekkarinen et al. 2000, Della Valle and Paprosky 2004, Ten Have et al. 2012).

Some smaller observational studies address bone defect size: in 86 patients with comparable femoral bone defects the choice of fixation has no certain influence on implant survival (Iorio et al. 2008), whereas uncemented revision stems conferred inferior implant survival compared with cemented revision stems in 209 patients with comparable femoral bone defects (Hernigou et al. 2015). However, both studies included different reasons for revision surgery, and the second study included both first-time and multiply revised patients.

Taken together, the available evidence on the optimal mode of revision stem fixation is hampered by small cohort sizes and lack of control groups (Berry et al. 1995, Iorio et al. 2008, Ornstein et al. 2009, Lakstein et al. 2010, Hernigou et al. 2015, Stigbrand and Ullmark 2017), there is a lack of information on indications underlying revision surgery (Iorio et al. 2008, Weiss et al. 2011, Hernigou et al. 2015), and, most importantly, in large register studies there is no information on the femoral bone defect sizes present at revision surgery (Weiss et al. 2011, Tyson et al. 2019). Our primary aim was therefore to compare the risk of re-revision of any component for any reason between uncemented and cemented stems in hip revision surgery with adjustment for preoperative femoral bone defect size in a large cohort of patients. Our secondary aims were to investigate whether uncemented and cemented revision stems were used in patients with different bone defect sizes, and to assess if the risk of stem re-revision, as well as risk of re-revision of any component due to aseptic loosening, dislocation, fracture, deep infection, and other reasons differed between the 2 fixation techniques.

## Patients and methods

This is an observational cohort study on patients registered in the SHAR, which collects data on patients who have undergone primary or revision THR since 1979 (Herberts et al. 1989), and the completeness is estimated at 92% (Swedish Hip Arthroplasty Register [SHAR] 2018).

The study cohort is based on a subgroup of patients from a previous study, evaluating stem fixation after revision THR, but without assessment of bone defect size (Tyson et al. 2019). Patients with first-time stem revision due to aseptic loosening performed 2006–2016 were identified in the SHAR. We chose 2006 as the starting point of our observation period in order to increase availability of preoperative radiographs because most hospitals archive their radiographs for a maximum of 15 years. We included revision surgeries performed at 9 orthopedic clinics that represented the national praxis in terms of the distribution of uncemented and cemented revision stem fixation according to the SHAR. During the investigated period 66% of revision stems were uncemented, and 34% cemented. We first selected clinics such that the most commonly used revision stems would be represented in our material. Second, only clinics with a minimal annual volume of 50 revisions were included in order to ascertain that low-volume clinics would not bias the results. A lower limit of 50 primary THRs has been reported as the minimal annual hospital volume to maintain a low revision rate in the Nordic countries, but we are unaware whether similar figures exist for the minimal annual volume of revision surgery (Glassou et al. 2016). Whenever patients were bilaterally revised during the study period, only the first revised side was included. Cement-in-cement revisions (Cnudde et al. 2017), in which the cement–bone interface is intact, were considered a separate technique and were therefore excluded from this analysis. Patients who were revised for reasons other than aseptic loosening at index surgery were also excluded in order to avoid bias introduced by the different outcomes after revisions performed due to infection, periprosthetic fracture, or dislocation. The variables method of revision stem fixation, stem type, date of re-revision, reason for re-revision, cup type at index revision, sex, age, comorbidity, date of death, and follow-up time were collected from the SHAR. Follow-up started on the day of surgery, and ended on the date of re-revision, death, emigration, or December 31, 2016, whichever came first.

### Bone defect size

The preoperative radiographs (pelvic view, anterior-posterior hip view, and lateral hip view) were retrieved and femoral bone defect size was independently assessed using the Paprosky classification (Della Valle and Paprosky 2004) by at least 2 researchers (YT, NM, or CH). The assessors were not aware of the inserted stem or the outcome when assessing the radiographs of the patients. The Paprosky classification (Table 1, see Supplementary data) was chosen because it offers substantial inter- and intraobserver reliability (Landis and Koch 1977, Brown et al. 2014), and is the classification most often used in previous studies comparing uncemented and cemented revision stems that include assessment of femoral bone defect size (Schmale et al. 2000, Iorio et al. 2008, Hernigou et al. 2015). Inter-observer reliability assessed using weighted Cohen’s kappa ranged between 0.65 and 0.90 in our study, and the intra-observer reliability was 0.90, which is assessed as substantial to almost perfect, according to the criteria of Landis and Koch (1977). In cases where classification by the 2 researchers did not agree, consensus was met under the guidance of the senior author (NH). Patients who had unclassifiable radiographs (for example due to osteosynthesis as a result of prior periprosthetic fracture), radiographs dating back over 2 years prior to index procedure, or unavailability of preoperative radiographs, were excluded.

**Table 2.** Characteristics of the study population. Values are count (%) unless otherwise specified

	Cemented	Uncemented
Factor	n = 266 (31)	n = 601 (69)
Mean age (SD)	74 (9)	72 (10)
Men	138 (52)	318 (53)
Mean follow-up, years (SD)	5 (3)	4 (3)
Diagnosis at primary THR		
Osteoarthritis	212 (80)	462 (77)
Fracture	14 (5)	34 (6)
Other	40 (15)	105 (17)
Type of revision		
Cup and stem revision	219 (82)	504 (85)
Stem revision only	47 (18)	90 (15)
Stem brand		
MP	0 (0)	291 (48)
Restoration	0 (0)	162 (27)
Wagner	0 (0)	78 (13)
Revitan	0 (0)	70 (12)
Lubinus SPII	123 (46)	0 (0)
Exeter	94 (35)	0 (0)
Spectron	49 (18)	0 (0)
Femoral bone defect size		
I	3 (1)	8 (1)
II	51 (19)	96 (16)
IIIA	170 (64)	449 (75)
IIIB	17 (6)	25 (4)
IV	25 (9)	23 (4)
Bone impaction grafting	125 (47)	23 (4)
Surgical approach		
Endofemoral	263 (99)	382 (64)
Transfemoral	3 (1)	219 (36)

### Bone impaction grafting and surgical approach

All surgical notes were retrieved and assessed by 1 of 2 authors (YT or CH). We searched for the terms “bone impaction grafting (of the femur),” or any detailed description thereof, the terms “endofemoral approach,” “transfemoral approach,” or “extended trochanteric osteotomy”, or any detailed description thereof. We also compared the preoperative assessment in the surgical notes with the information in the register database in order to ascertain that index revisions were correctly classified as being due to aseptic loosening and whether they were indeed first-time revisions. Only 2% of the procedures were incorrectly classified as aseptic loosening or first-time revisions, and these were excluded.

### Outcomes

Primary outcome was re-revision of any component for any reason, i.e., only cup, head, or stem revision, any combination thereof, or extraction of the prosthesis. Secondary outcomes were percentage of preoperative femoral bone defect sizes in both fixation groups, re-revision of the stem for any reason, and reasons for re-revision.

### Confounders

The confounders age at index surgery, sex, and femoral bone defect size were included in multivariable regression models. We chose confounders by using a strict epidemiological approach in which only those factors that we believed would affect both exposure and outcome were deemed confounders. To illustrate our thought process, we constructed a directed acyclic graph (Figure 1, see Supplementary data) with the use of the dagitty.net online software. Use of surgical approach or of femoral bone impaction grafting were considered mediators, concomitant cup revision, and femoral head size were considered as having their own causal pathway, and all these variables were thus not defined as confounders. Since comorbidity as measured by ASA grade was first introduced in 2008 in the register and therefore not complete in our dataset, comorbidity was adjusted for only in a subgroup of patients. Bone defect size as confounder was divided into 3 groups: group 1 (Paprosky I and II), group 2 (Paprosky IIIA), and group 3 (Paprosky IIIB and IV), in order to obtain sufficient numbers of patients in each group.

**Figure 2.**
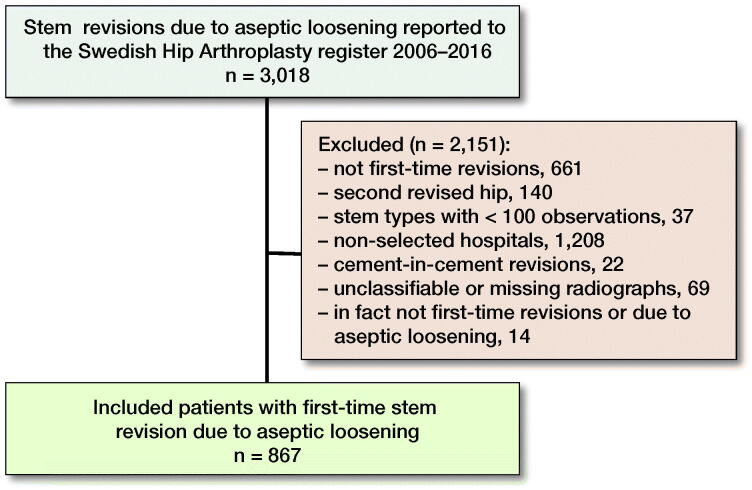
Flow chart of included patients.

### Statistics

Demographics including femoral bone defect size were described with percentages, means, and standard deviations. Unadjusted implant survival was estimated using the Kaplan–Meier method. Risks of re-revision of any component or of the stem were expressed as hazard ratios (HR) with 95% confidence intervals (CI) and estimated by fitting multivariable Cox regression models adjusted for the confounders described above. Since the unadjusted cumulative hazard function for the endpoint re-revision for any reason deviated considerably from the assumption of proportionality, the follow-up time was divided into 2 time periods at the point where the curves began to converge. The maximum difference between the survival curves was calculated to 2.95 years and for reasons of readability 3 years was chosen as the dividing point. Thus, the 1st time period consisted of the 1st 3 years after index surgery and the 2nd time period ranged from the 4th to the 8th year after index surgery. Schoenfeld residuals were calculated in order to assess whether model assumptions were met.

In order to assess the risk of re-revision due to aseptic loosening, dislocation, periprosthetic fracture, infection, or other reasons, cumulative incidence functions and subdistribution HRs with 95% CI were calculated using a competing risks model according to Fine and Gray (1999). All reasons for re-revision and death were considered competing events.

Thorough sensitivity analyses were conducted (see Supplementary data).

R Statistical Software (R version 3.4.3, 2017-11-30, R Foundation for Statistical Computing, Vienna, Austria), packages: haven, pastecs, knitr, Epi, rms, Gmisc, magrittr, tidyverse, irr, lpSolve, kableExtra, ggplot2, cmprsk, crrSC, scales, and reshape2 were used for the calculations (R Core Team 2017).

### Ethics, funding, and potential conflicts of interest

Ethical approval was obtained from the Regional Ethics Review Board in Uppsala, Sweden (decision 2018/076). Financial support was received from the Health Care Committees in Region Uppsala and Region Västra Götaland. No competing interests were declared.

## Results

A cohort of 867 patients was included in this study (Figure 2). The study cohort was divided into 2 groups of patients, 1 operated on with uncemented (n = 601, 69%) and 1 with cemented revision stems (n = 266, 31%). The use of uncemented revision stems was slightly higher in the second half of the study period (Figure 3, see Supplementary data). The mean follow-up time was 4.5 (SD 3.0) years and the mean age at index surgery was 72 (SD 10) years. The groups had similar sex distribution, but patients who received uncemented revision stems were on average younger at the time of surgery and had a shorter mean follow-up time. Most patients in both fixation groups had medium size femoral bone defects prior to surgery, but fewer of those who received uncemented stems had large bone defects (8%) compared with patients with cemented stems (15%) prior to index surgery (Table 2). In the cemented group, bone impaction grafting was performed equally often when large bone defects were present as cementation alone (Table 3, see Supplementary data). Of the clinics that performed cemented fixation, most clinics tended to use bone impaction grafting in either all or none of the cases (Figure 4, see Supplementary data).

### Re-revision of any component for any reason

**Figure 5.**
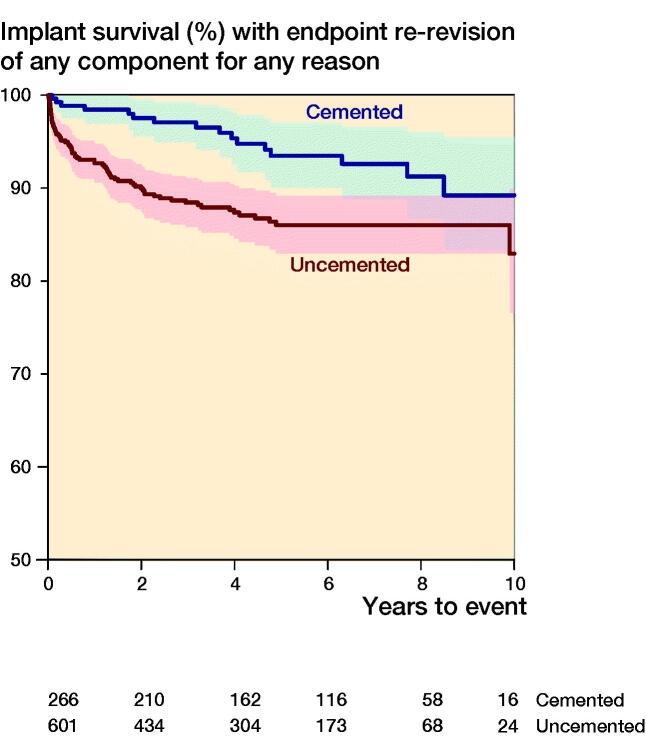
Unadjusted implant survival with endpoint re-revision of any component.

The unadjusted 10-year implant survival with re-revision of any component for any reason was lower after use of uncemented revision stems compared with cemented stems (83%, CI 77–99 versus 89%, CI 83–95, Figure 5), but with overlapping CIs. During the first 3 years after index surgery, we attained a higher estimate for the adjusted risk of re-revision of any component for any reason in patients with uncemented compared with those with cemented revision stems (HR 4, CI 2–9). Between the 4th and 8th year, the adjusted risk of re-revision of any component for any reason was similar in both groups (HR 0.5, CI 0.2–1.4).

### Stem re-revision for any reason

The unadjusted 10-year implant survival with stem re-revision for any reason was lower after use of uncemented revision stems compared with cemented stems (90%, CI 87–93 versus 94%, CI 90–98), but with overlapping CIs. The adjusted risk of stem re-revision for any reason was higher for patients with uncemented compared with those with cemented revision stem fixation during the first 3 years after index surgery (HR 6, CI 2–15). Between the 4th and 8th year, the adjusted risk of stem re-revision for any reason was lower after uncemented compared with cemented revision stem fixation (HR 0.3, CI 0.1–1.0).

### Risk of re-revision for different reasons

**Figure 6.**
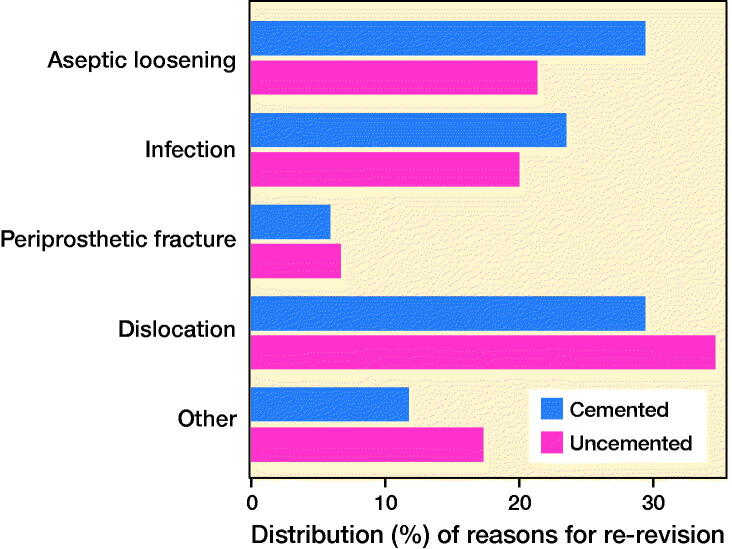
Reasons for re-revision.

Re-revisions of any component after the use of uncemented revision stem fixation were most often performed due to dislocation (35%), whereas re-revisions after cemented revision stem fixation were most often performed due to aseptic loosening (30%) or dislocation (30%, Figure 6). The risk of re-revision due to dislocation was higher for uncemented compared with cemented revision stems, both during the first 3 years and during the 4th to 8th year after index surgery (HR 5, CI 1.2–23 and HR 3, CI 1.0–9). There was no statistically significant difference in risk of re-revision due to aseptic loosening, deep infection, fracture, or other reasons, between uncemented and cemented revision stems (Table 4).

**Table 4.** Subdistribution hazard ratios (HR) for re-revision of uncemented compared with cemented revision stems, estimated with the Fine and Gray method

	Years after index surgery	
	0–3	4–8
Reason for re-revision	HR (95% CI)	HR (95% CI)
Aseptic loosening	2 (0.5–9)	1 (0.5–4)
Deep infection	3 (0.5–11)	2 (0.5–5)
Periprosthetic fracture	Too few events	3 (0.3–28)
Dislocation	5 (1.2–23)	3 (1–9)
Other reasons	6 (0.8–51)	3 (0.7–15)

Aseptic loosening, deep infection, periprosthetic fracture, dislocation, other reasons for re-revision, and death were considered competing events. The regression was adjusted for age, sex, and femoral bone defect size (aggregating defects into 3 main groups along Paprosky classes I + II, IIIA, IIIB + IV)

## Discussion

Uncemented stems are gradually replacing cemented stems in hip revision surgery in Sweden (Swedish Hip Arthroplasty Register [SHAR] 2015) but there is no study comparing the 2 fixation concepts with a sample size above 209 patients including assessment of femoral bone defect size (Iorio et al. 2008, Weiss et al. 2011, Hernigou et al. 2015, Tyson et al. 2019). Our study is the largest comparative study on uncemented and cemented revision stems with stringent assessment of preoperative femoral bone defect size, and our main finding is that although both concepts have satisfactory medium-term outcomes, the risk of re-revision for any reason is considerably higher after the use of uncemented revision stems during the first 3 years. For the remainder of the observation period the risk seems similar.

Even though the choice of fixation method is ultimately surgeon-dependent, the increased use of uncemented revision stems might be due to the fact that modular stems offer the option of distal anchoring within intact bone, and several opportunities exist to vary the proximal part in order to achieve optimal soft tissue tension, anteversion, and offset (Weiss et al. 2009). Another explanation could be that primary cemented stems have higher risk of aseptic loosening compared with uncemented (Mäkelä et al. 2008, 2011), which is why it would be logical to assume that uncemented revision stems would decrease the risk of subsequent revisions in the case of aseptic loosening, a phenomenon that has been observed among revision stems in previous studies (Weiss et al. 2011, Tyson et al. 2019).

Previous register-based studies state that uncemented revision stems fail more frequently during the early postoperative period but might confer a lower risk of loosening in the long term (Weiss et al. 2011, Tyson et al. 2019). Our present study supports that uncemented revision stems fail more frequently during the early postoperative years, and it adds to the cited studies in that we were able to adjust for femoral bone defect size. In fact, fewer patients with uncemented stems had large femoral bone defects prior to surgery compared with patients receiving cemented stems, thus bone defect size seems not to be the main reason for difference in implant survival, which has been suggested (Weiss et al. 2011). No particular stem model within each fixation group deviated considerably from the mean risk of re-revision within each group (data not shown).

One could question our results given that Swedish surgeons are more accustomed to using cemented primary implants. However, in our previous study we stratified our results based on the year of implantation as a proxy for becoming accustomed to the uncemented fixation technique, and no major differences were observed (Tyson et al. 2019). In a cohort study with preoperative femoral bone defect assessment that comprised 209 patients, 21% of the uncemented revision stems were re-revised at 5 years, in contrast to none of the cemented revision stems (Hernigou et al. 2015). However, the risk of cardio-pulmonary events after surgery but not re-revision was the primary outcome in that study. In an observational, matched cohort study including 86 patients, uncemented and cemented revision stems are comparable in terms of implant survival at 5 years (Iorio et al. 2008). However, only low-grade femoral bone defects corresponding to Paprosky classes I to II were studied, which limits the generalizability of these findings. Although a study by Schmale et al. (2000) demonstrated superior survivorship of uncemented stems, the comparator group was pre-coated cemented stems, which are no longer used due to increased risk of early failure (Ong et al. 2002). Previous studies have suggested that uncemented revision stems should be used in younger patients, but cemented ones in the elderly (Weiss et al. 2011, Tyson et al. 2019). Our study does not support this assumption; however, the events in each age group were few in number, thus limiting the conclusions to be drawn, and one can argue that the elderly with short life expectancy would benefit from the decreased risk of early complications associated with cemented stem fixation. Further, our results are applicable only to revisions caused by aseptic loosening, and further research is necessary to investigate outcomes after revisions due to infection, periprosthetic fracture, dislocation, and other causes.

We have previously reported similar results concerning reasons for re-revision after revision THR in a register-based cohort, but without assessment of femoral bone defect sizes (Tyson et al. 2019). Our study lends support to the theory that the different failure mechanisms observed in uncemented and cemented revision stems are at least in part analogous to the failure mechanisms of these 2 fixation principles in primary THR, where dislocation as an early complication is more frequently observed after uncemented stem fixation (Hailer et al. 2010, Pedersen et al. 2014, Gromov et al. 2015, Tyson et al. 2019). The increased incidence of re-revisions due to dislocation in patients who received uncemented revision stems may in part be the result of stem subsidence, but without evaluation of serial radiographs up to the re-revision such a connection cannot be confirmed (Malchau et al. 1997, Paprosky et al. 1999, Mardones et al. 2005, Ström et al. 2006, Lakstein et al. 2010, Hernigou et al. 2015, Klein et al. 2019). Dislocation is associated with smaller head sizes (Hailer et al. 2012a); however, in our study uncemented stems on average had larger head sizes, which is why this phenomenon does not explain our observation. Further, without any follow-up visits we could not evaluate whether abduction impairment differed between the groups. It should be emphasized that we did not record the rate of dislocations but only the rate of re-revisions due to dislocation, and it might be that the threshold to re-revise the proximal part of a modular uncemented revision stem is lower than before re-revising a well-cemented revision stem.

Aseptic stem loosening is a late complication, and after primary THR loosening is more common after the use of cemented than after that of uncemented stems (Mäkelä et al. 2008, 2011, Pedersen et al. 2014, Gromov et al. 2015). The increased risk of aseptic loosening among cemented revision stems could be due to longer follow-up of this group. It is also suggested that aseptic loosening of cemented stems results from debonding of the cement from the femoral canal (Sundfeldt et al. 2006), and the risk of its occurrence could increase with increasing magnitude of bone defects, but this phenomenon was not observed in our study. The technique of bone impaction grafting was introduced to restore bone stock and to facilitate cementation in a femoral canal devoid of trabecular bone, and modern bone impaction techniques yield promising results (Ornstein et al. 2009, Howie et al. 2010, Wilson et al. 2016, Stigbrand and Ullmark 2017). In our study bone impaction grafting in combination with cementation did not reduce the risk of re-revision compared with cementation alone, even though the use of bone impaction grafting was performed equally often in patients with large bone defects as was cementation alone. Further, when we restricted the cohort to patients that did not receive bone impaction grafting, there was no statistically significant difference when compared with the estimates derived from the main analysis. However, since we only have short- to medium-term follow-up, the full effect of bone impaction grafting might not have been observed in our study. It should be noted that although cemented revision stems were more often re-revised due to aseptic loosening than uncemented stems, there was no statistically significant difference in the risk of re-revision due to aseptic loosening between the 2 fixation methods in the competing risk analysis. However, a lack of statistically significant differences between groups, with confidence intervals that include 1.0, does not imply evidence of absence of differences between groups.

The use of dual mobility cups at index surgery protected against re-revision due to dislocation among uncemented revision stems in our study, a phenomenon reported by other authors as well, in both the primary and revision setting (Hailer et al. 2012b, Mohaddes et al. 2017, Jobory et al. 2019, Kreipke et al. 2019). However, to study dual mobility cups was not the primary aim of our study so these results should be interpreted with some caution. There was no statistically significant difference in the incidence of periprosthetic fracture, even though previous authors report increased risk of periprosthetic fracture after use of uncemented stems (Hailer et al. 2010, Mäkelä et al. 2014, Thien et al. 2014, Gromov et al. 2015). One explanation for this could be the use of bone impaction grafting in conjunction with cementation; bone impaction grafting has been reported as a risk factor for perioperative periprosthetic fracture (Ornstein et al. 2002). In our study we did not investigate perioperative complications, only subsequent re-revisions. Further, in our study, most patients had the primary diagnosis osteoarthritis, but in a subgroup analysis on patients with a diagnosis of femoral neck fracture at primary arthroplasty surgery (n = 48) no patient was re-revised due to periprosthetic fracture.

### Strengths and limitations

To our knowledge, our study represents the largest comparison of uncemented and cemented revision stems with stringent assessment of femoral bone defect size at the time of revision surgery. We performed several sensitivity analyses that supported our main results, but the study has its limitations.

1st, the follow-up time was only medium-term, thus possibly underestimating the incidence of aseptic loosening, which could positively bias the outcome after cemented revision stem fixation, while similarly underestimating the potentially beneficial effects of bone impaction grafting in the long-term.

2nd, bone defects in the more advanced stages of the Paprosky classification were scarce, which could imply that our conclusions are mainly valid for small to medium sized femoral bone defects. Although bone defect size was controlled for in the main analyses and sensitivity analyses stratified on bone defect size indicated similar results, one should be cautious when interpreting our results. It cannot be concluded, based on our data, that bone impaction grafting or uncemented stems should not be used in extensive femoral bone defects as we lack both statistical precision and long-term data for these scenarios.

3rd, the generalizability of our study can be questioned given that only half of the revision patients operated on within the time frame of this study were included, also excluding patients from low-volume institutions. On the other hand, outcomes after first-time revisions due to aseptic loosening in all revisions in Sweden but without assessment of preoperative radiographs were very similar (Tyson et al. 2019), indicating that that the results of this present study are valid.

4th, given that Sweden is a “cementation country,” surgeons’ learning curves could possibly influence the results after uncemented stems, for example if the surgeon is afraid to use a wider uncemented stem to achieve more stable fixation that prevents subsidence. However, in a previous study by the present authors on revision THR and fixation technique (Tyson et al. 2019), we evaluated whether year of implantation as proxy for learning a new technique influenced the results, and no influence was observed.

5th, even though bone impaction grafting was a variable collected from the surgical notes, bone impaction grafting is a surgical technique with certain principles, and it is difficult to know to what extent these principles were adhered to. In addition, as with all study designs, the results and conclusions are more applicable to the studied population; however, with an assessment of preoperative bone defect size, we believe the conclusions may be somewhat more generalizable. On the other hand, depending on their surgical experience with cementation, the present results may not apply to all countries.

Finally, the validity of register data can always be questioned, but completeness is estimated to be 92% for revision THR in the SHAR (Swedish Hip Arthroplasty Register [SHAR] 2018). Further, variables such as bone impaction grafting and surgical approach were directly collected from the surgical notes, and surgical notes are routinely sent to the register to assess the correctness of data. In our study, only 2% of the patients had incorrectly classified data and these were excluded.

## Conclusions

This large, register-based study with stringent assessment of femoral bone defect sizes indicates that uncemented revision stems are not used in more complex revision situations, but that they still confer an increased early risk of re-revision after revision THR, mostly due to dislocations occurring during the first postoperative years. Cemented stem fixation is a good option in hip revision surgery, and our findings do not support the declining use of this fixation technique. We advocate the use of cemented fixation in older patients with short life expectancy who will benefit from reduced risk of early complications, even though the ultimate choice of stem must be individualized. The different reasons for failure should be considered when counselling patients prior to these procedures, and measures should be taken to avoid the specific failure mechanisms associated with the different fixation methods.

## Supplementary Material

Supplemental Material

## Supplementary data

Table 1 and 3 and Figures 1, 3, 4, 7, and 8 are available in the online version of this article, as well as methodological description and results of the sensitivity analyses, http://dx.doi.org/10.1080/17453674.2020.1846956
