# Supplementary material for: Uncemented or cemented stems in first-time revision total hip replacement? An observational study of 867 patients including assessment of femoral bone defect size
Source: Acta Orthop. 2020 Nov 12;92(2):143–50. doi: 10.1080/17453674.2020.1846956 (PMC8159203; doi:10.1080/17453674.2020.1846956)
Supplement: Supplemental Material [file IORT_A_1846956_SM8898.pdf]

Supplementary data

Table 1. The Paprosky classification

Type I	Minimal metaphyseal bone loss
Type II	Extensive metaphyseal bone loss and an intact diaphysis
Type IIIA	Extensive metadiaphyseal bone loss and a minimum of 4 cm of intact cortical bone in the diaphysis
Type IIIB	Extensive metadiaphyseal bone loss and < 4 cm of intact cortical bone in the diaphysis
Type IV	Extensive metadiaphyseal bone loss and a non-supportive diaphysis

Sensitivity analyses

1st, to assess whether dual mobility cups reduced the risk of dislocation, which was found to be increased among patients operated on with uncemented revision stems, the risk of re-revision due to dislocation was compared between patients who received dual mobility cups, and those who received regular cups, in a subcohort of patients restricted to uncemented revision stems (n = 601). 2nd, to investigate the association of surgical approach with risk of re-revision, a subcohort of patients with only uncemented revision stems (n = 601) was analyzed, comparing unadjusted and adjusted stem survival after endofemoral compared with transfemoral approaches. In addition, to exclude the potential impact of using transfemoral approaches on implant survival, analyses were restricted to a subcohort of patients operated by an endofemoral approach (n = 645). 3rd, in order to investigate the outcome after femoral bone impaction grafting, a subcohort of patients who had undergone only cemented femoral revision (n = 266) was analyzed, comparing unadjusted and adjusted stem survival between patients who received femoral bone impaction grafting or not. In addition, to exclude the potential benefits of using femoral bone impaction grafting in conjunction with the use of cemented revision stems, analyses were restricted to a subcohort of patients not including femoral bone impaction grafting (n = 719). 4th, to assess whether age at implantation affected risk of re-revision, event rate per 100 person years was calculated for uncemented and cemented revision stems depending on age. 5th, to adjust for comorbidity, analyses were repeated for a subcohort of patients operated on from 2009, for whom information on ASA class was reported to the SHAR (n = 645). Lastly, to investigate how uncemented and cemented revision stems perform in different bone defect sizes, analyses were stratified by femoral bone defect sizes, grouped into the 2 main groups with Paprosky class I, II, and IIIA (n = 777) and those with Paprosky class IIIB and IV (n = 90). The same confounders used in our primary analyses were used in the sensitivity analyses.

Table 3. Bone impaction grafting in the cementation group, depending on bone defect size. Values are count (%)

Bone impaction grafting	Femoral bone defect size I + II	IIIA	IIIB + IV
Yes	15 (28)	89 (48)	21 (50)
No	39 (72)	81 (52)	21 (50)

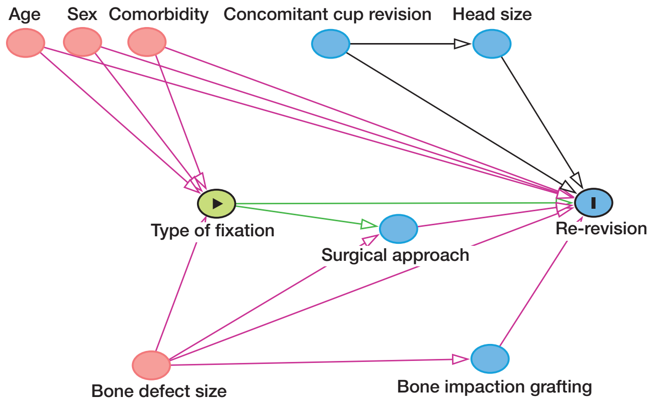

Figure 1. The DAG used to select confounders. Red circles with red arrows indicate variables used in the statistical model. Blue circles with red arrows indicate mediators. Blue circles with black arrows indicate another causal pathway.

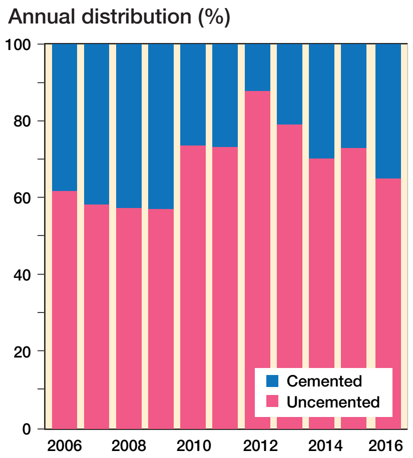

Figure 3. Type of revision stem used by year of inclusion.

 Acta Orthopaedica 2020; 91 (DOI: 10.1080/17453674.2020.1846956)

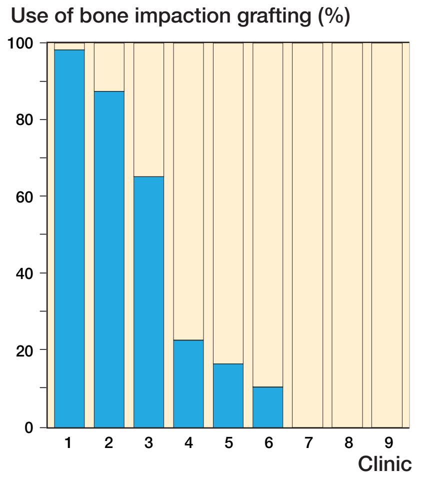

Figure 4. Use of bone impaction grafting per clinic in conjunction with a cemented stem.

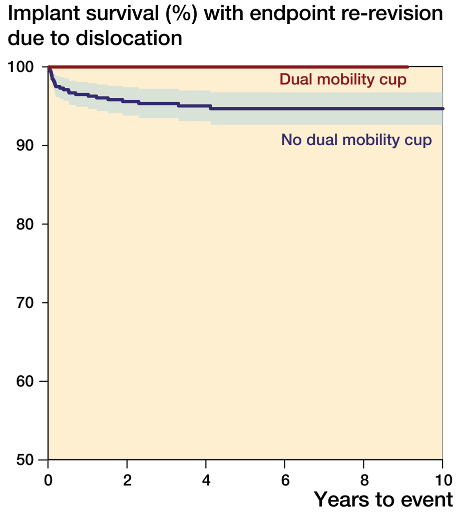

At risk

Dual mobility cup	532	391	279	161	67	24
No dual mobility cup	69	43	25	12	1	0

Figure 7. Unadjusted implant survival with endpoint re-revision due to dislocation with exposure dual mobility cups.

Sensitivity analyses

Dual mobility cups inserted in conjunction with an uncemented revision stem conferred a reduced risk of re-revision due to dislocation (Figure 7, see Supplementary material). The use of transfemoral surgical approaches was not associated with a considerably different risk of re-revision of any component for any reason compared with endofemoral approaches. Similarly, there was no difference in the risk of re-revision for any reason when the use of femoral bone impaction grafting was compared with cementation alone. Age at implantation did not alter the results. Further sensitivity analyses did not differ from our primary results, including when the cohort was restricted to patients who did not receive bone impaction grafting, or when the cohort was divided into small or large bone defect sizes.
